# Stochastic *H*_∞_ Filtering of the Attitude Quaternion †

**DOI:** 10.3390/s24247971

**Published:** 2024-12-13

**Authors:** Daniel Choukroun, Lotan Cooper, Nadav Berman

**Affiliations:** Mechanical Engineering Department, Ben-Gurion University of the Negev, Beer Sheva 84105, Israel; lotanco@gmail.com (L.C.);

**Keywords:** uncertain sensor variance, stochastic *H*_∞_ filtering, quaternion estimation

## Abstract

Several stochastic $H_{\infty }$ filters for estimating the attitude of a rigid body from line-of-sight measurements and rate gyro readings are developed. The measurements are corrupted by white noise with unknown variances. Our approach consists of estimating the quaternion while attenuating the transmission gain from the unknown variances and initial errors to the current estimation error. The time-varying $H_{\infty }$ gain is computed by solving algebraic and differential linear matrix inequalities for a given transmission threshold, which is iteratively lowered until feasibility fails. Thanks to the bilinear structure of the quaternion state-space model, the algorithm parameters are independent of the state. The case of a gyro drift is addressed, too. Extensive Monte-Carlo simulations show that the proposed stochastic $H_{\infty }$ quaternion filters perform well for a wide range of noise variances. The actual attenuation, which improves with the noise variance and is worst in the noise-free case, is better than the guaranteed attenuation by one order of magnitude. The proposed stochastic $H_{\infty }$ filter produces smaller biases than nonlinear Kalman or unscented filters and similar standard deviations at large noise levels. An essential advantage of this $H_{\infty }$ filter is that the gains are independent of the quaternion, which makes it insensitive to modeling errors. This desired feature is illustrated by comparing its performances against those of unmatched nonlinear optimal filters. When provided with too high or too low noise variances, the multiplicative Kalman filter and the unscented quaternion filter are outperformed by the $H_{\infty }$ filter, which essentially delivers identical error magnitudes.

## 1. Introduction

The attitude quaternion ([1], p. 758) is a very popular spacecraft attitude parametrization, whose mathematical modeling and filtering have been ongoing topics of research for more than four decades [2]. Numerous successful quaternion stochastic estimators have been developed in the realm of optimal filtering, e.g., [3,4,5,6,7,8] and ([9], Ch. 6) for an in-depth survey. An inherent drawback of the optimal filtering approach is its sensitivity to the model noise variance. Although adapting the filter gain computations might provide satisfactory performances in some cases, like adaptive process noise estimation in [6] and uncompensated random biases in [10,11], the designer might prefer a less sensitive approach: rather than trying to estimate the unknown parameters, the filter will attenuate their impact on the estimation error for a given transmission level that should be as small as possible. This general approach was followed in [12,13,14] for spacecraft attitude determination. In [12], an $H_{\infty }$ estimator of the spacecraft quaternion and gyro biases was developed. In [13,14], extended $H_{\infty }$ filters were applied to spacecraft attitude determination and gyro calibration by processing space-borne telemetry of the CBERS-2 satellite, including outputs from rate gyros, two sun sensors, and an Earth sensor. The filters produced estimates of Euler angles and the quaternion, respectively, along with estimates of the gyro biases. Their performance compared favorably with those of standard extended Kalman filters. Reference [15] presented an invariant extended $H_{\infty }$ filter for attitude and position estimation using Lie groups algebra, which conveniently produces state-independent Jacobians at any linearization point. All of the above works are rooted in the deterministic $H_{\infty }$ estimation theory. In that realm, the measurement and process noises, and the initial errors, are modeled as energy-finite disturbances, a.k.a. $L_{2}$ or $l_{2}$ signals.

A theory of stochastic $H_{\infty }$ filtering and control for nonlinear systems was introduced in [16,17] based on dissipativity theory. The stochastic framework has several advantages over the deterministic one. It enables the treatment of random parameters in the measurement and process equations; it enables the treatment of white noises in the process and measurement equations; it alleviates the requirement for perfect knowledge of the white noise variance; conceptually, it includes the deterministic case as a particular case. These are significant theoretical departures from the deterministic setting and they have significant practical aspects as well. In our work, the state-space model parameters are random because they are functions of gyroscopes and vector measurements. While the deterministic theory is not built to cope with random parameters, the stochastic theory does by considering an averaged version of the $L_{2}$ gain property. The developments involve a conditional expectation operator given the available information, which enables processing current observations. Using white noise as a modeling step for process and sensing errors is common in engineering applications. A famous example in aeronautics is the Dryden Wind Turbulence model where continuous gusts are modeled as stochastic processes with several distinct power spectral densities. The case for modeling measurement errors as random noises is even stronger, hence, the need for a stochastic $H_{\infty }$ framework that encompasses white noise inputs. Furthermore, the proposed approach alleviates the need for perfect knowledge of the noise variances.

In this work, stochastic $H_{\infty }$ quaternion filters are developed in the realm of the theory introduced in [16,17] to estimate attitude from vector and gyroscope observations. We assume that a time-varying line-of-sight (LOS) signal is continuously acquired, that a triad of body-mounted gyros provides a measurement of the spacecraft’s angular velocity, and that both measurement processes are corrupted by additive white noise. The case of a gyro bias is addressed through Brownian motion modeling. All white noise variances are assumed to be unknown. A distinctive feature of our modeling and filtering approach is that the plant equations admit white noise as inputs and that their variances are assumed to be finite-energy perturbations. The stochastic filter aims at estimating the quaternion while attenuating these perturbations. This article is a revised and expanded version of the paper [18]. Here, it is extended to include the development of an $H_{\infty }$ filter for quaternion and gyro bias estimation, along with extensive Monte-Carlo simulations for validation.

Several filters are developed: first, the gyro noise variance is the sole unknown, then both the gyro and the LOS noise variances are unknown, and, finally, the case of biased gyro measurements is considered. The state multiplicative nature of the errors in both the process and measurement provides a useful structure. The proposed approach avoids linearization by exploiting a pseudo-linear quaternion plant model, introduced in [6], extended to continuous-time stochastic settings, and further analyzed in [7,8]. The filter implementation requires solving differential linear matrix inequalities that do not depend on the state estimate. Extensive Monte-Carlo simulations were run to evaluate the novel filter’s performances and to compare them with those of a quaternion multiplicative extended Kalman filter [3], an unscented quaternion filter [5], and the quaternion Kalman filter in [7].

Section 2 presents the quaternion $H_{\infty }$ filters for the case of drift-free rate gyros. Section 3 is concerned with the non-zero drift case. Section 4 presents the results of Monte-Carlo simulations. Conclusions are proposed in Section 5.

## 2. Quaternion Stochastic $H_{\infty }$ Filtering

### 2.1. Problem Statement

For simplicity and clarity, drift-free gyroscopes are treated first. Consider the following Itô stochastic differential equation (SDE) for the attitude quaternion, and the associated measurement equation [7]:

(1) $$\begin{array}{cc} \; & dq_{t}=\frac{1}{2}\left(\Omega_{t}-\frac{3\sigma_{\epsilon }^{2}}{4}I_{4}\right)q_{t}dt-\frac{1}{2}\Xi \left(q_{t}\right)\sigma_{\epsilon }{d\beta }_{t};\;q_{t}\left(0\right)\overset{a.e.}{=}q_{0};\;t\in \left[0,T\right] \end{array}$$

(2) $$\begin{array}{cc} \; & dy_{t}=H_{t}q_{t}dt-\frac{1}{2}\Xi \left(q_{t}\right)\sigma_{b}{d\eta }_{t}\; \end{array}$$

where $q_{t}\in R^{4}$ denotes the attitude quaternion, $\Omega_{t}\in R^{4\times 4}$ is the following matrix function of the measured angular velocity $\omega_{t}$,

(3) $$\begin{array}{cc} \; & \Omega_{t}=\left[\begin{array}{cc} -\left[\omega_{t}\times \right] & \omega_{t}\\ -\omega_{t}^{T} & 0 \end{array}\right] \end{array}$$

where $\omega_{t}\in R^{3}$, which is acquired by a triad of body-mounted gyroscopes, is corrupted by an additive standard Brownian motion, $\beta_{t}\in R^{3}$, with infinitesimal independent increments ${d\beta }_{t}$, such that $E\left\{{d\beta }_{t}{d\beta }_{t}^{T}\right\}=I_{3}dt$. The parameter $\sigma_{\epsilon }$ denotes the standard deviation of the gyro noise $\beta_{t}$. The drift term in Equation (1) features a damping term in $\sigma_{\epsilon }$ that ensures mean-square stability of the process $q_{t}$ (see [7,8]). Under the conditions governing Equation (1), it was shown in [7] that the trace of $E\{q_{t}q_{t}\}$ is invariant as *t* increases, and in [8] that the steady-state of $E\{q_{t}q_{t}\}$ as *t* tends to infinity is the scaled identity matrix $\frac{1}{4}I_{4}$. Equation (2) is a continuous-time quaternion measurement equation where the measurement value is identically zero [6]. Hence,

(4) $$\begin{array}{c} dy_{t}=0_{4\times 1} \end{array}$$

and the measurement matrix $H_{t}\in R^{4\times 4}$ is constructed from LOS measurements. Let $b_{t}\in R^{3}$ and $r_{t}\in R^{3}$ denote the projections of a measured LOS in the spacecraft body frame axes and a reference inertial frame, respectively, then $H_{t}$ is computed as follows

(5) $$\begin{array}{cc} \; & s_{t}=\frac{1}{2}\left(b_{t}+r_{t}\right) \end{array}$$

(6) $$\begin{array}{cc} \; & d_{t}=\frac{1}{2}\left(b_{t}-r_{t}\right) \end{array}$$

(7) $$\begin{array}{cc} \; & H_{t}=\left[\begin{array}{cc} -\left[s_{t}\times \right] & d_{t}\\ -d_{t}^{T} & 0 \end{array}\right]\; \end{array}$$

The matrix, $\Xi (q_{t})$, which appears both in the process and measurement multiplicative noises, is the following linear matrix function of the quaternion $q_{t}={\left[e_{t}^{T}q_{t}\right]}^{T}$:

(8) $$\Xi \left(q_{t}\right)=\left[\begin{array}{c} q_{t}I_{3}+\left[e_{t}\times \right]\\ -e_{t}^{T} \end{array}\right]$$

The measurement noise is modeled as a standard Brownian motion, $\eta_{t}\in R^{3}$, multiplied by the parameter $\sigma_{b}$. Assume that $\sigma_{\epsilon }$ and $\sigma_{b}$ are unknown, possibly time-varying random parameters, and that there is no prior knowledge of their possible values or bounds. The filtering problem consists of estimating the quaternion $q_{t}$ in the presence of unknown and random $\sigma_{\epsilon }$ and $\sigma_{b}$ and is formulated as a disturbance attenuation problem. The following estimator is considered:

(9) $$\begin{array}{cc} \; & d{\hat{q}}_{t}=\frac{1}{2}\Omega_{t}{\hat{q}}_{t}dt+K\left({\hat{q}}_{t}\right)\left(dy_{t}-H_{t}{\hat{q}}_{t}dt\right) \end{array}$$

(10) $$\begin{array}{cc} \; & \hat{q}\left(0\right)={\hat{q}}_{0}\; \end{array}$$

The Itô correction term, $\frac{3\sigma_{\epsilon }^{2}}{4}$, is dropped for simplicity. It is not required for the filter development as the dynamics of the filter estimate and estimation error, $\left\{{\hat{q}}_{t},{\tilde{q}}_{t}\right\}$, are designed to be stable in the mean-square sense independently from that correction [16]. Let ${\tilde{q}}_{t}$ denote the additive quaternion estimation error, i.e.,

(11) $$\begin{array}{cc} \; & {\tilde{q}}_{t}=q_{t}-{\hat{q}}_{t} \end{array}$$

Given a scalar γ>0, a gain process $K\left({\hat{q}}_{t}\right)\in R^{4\times 4}$ is sought, such that the following $H_{\infty }$ criterion is satisfied:

(12) $$\begin{array}{c} E\{\int_{0}^{T}∥{\tilde{q}}_{t}{∥}^{2}dt\}\leq \gamma^{2}\;E\{∥{\tilde{q}}_{0}{∥}^{2}+\int_{0}^{T}∥v_{t}{∥}^{2}dt\} \end{array}$$

under the constraints (1) and (2), and where $v_{t}$ denotes the augmented process of admissible disturbance functions. Whenever Equation (12) is true, the so-called $L_{2}$-gain property from $\left\{{\tilde{q}}_{0},v_{t}\right\}$ to ${\tilde{q}}_{t}$, is satisfied for 0≤t≤T. Two cases for v will be considered in the following: $v=\sigma_{\epsilon }$ and $v=\{\sigma_{\epsilon },\sigma_{b}\}$ and significant differences will be highlighted.

### 2.2. Augmented Stochastic Process

Following standard techniques, the following augmented process is defined:

(13) $$\begin{array}{cc} \; & q_{t}^{a}\;\overset{▵}{=}\;\left[\begin{array}{c} {\hat{q}}_{t}\\ {\tilde{q}}_{t} \end{array}\right] \end{array}$$

The design model for the process $q_{t}$ obeys the following equation

(14) $$\begin{array}{cc} \; & dq_{t}=\frac{1}{2}\Omega_{t}q_{t}dt-\frac{1}{2}\Xi \left(q_{t}\right)\sigma_{\epsilon }{d\beta }_{t}\; \end{array}$$

The estimator’s equation is given by Equation (9), i.e.,

(15) $$\begin{array}{cc} d{\hat{q}}_{t} & =\frac{1}{2}\Omega_{t}{\hat{q}}_{t}dt+{\hat{K}}_{t}\left(dy_{t}-H_{t}{\hat{q}}_{t}dt\right)\\ \; & =\left[\frac{1}{2}\Omega_{t}-{\hat{K}}_{t}H_{t}\right]{\hat{q}}_{t}dt\; \end{array}$$

where ${\hat{K}}_{t}$ denotes $K({\hat{q}}_{t})$. Using Equations (2), (14) and (15) yields the SDE for the estimation error:

(16) $$\begin{array}{cc} d{\tilde{q}}_{t} & =\left[\frac{1}{2}\Omega_{t}-{\hat{K}}_{t}H_{t}\right]{\tilde{q}}_{t}dt-\frac{1}{2}\Xi \sigma_{\epsilon }{d\beta }_{t}+\frac{1}{2}{\hat{K}}_{t}\Xi \sigma_{b}{d\eta }_{t}\\ \; & =\left[\frac{1}{2}\Omega_{t}-{\hat{K}}_{t}H_{t}\right]{\tilde{q}}_{t}dt-\frac{1}{2}\sum_{i=1}^{3}\Xi_{Ci}\sigma_{\epsilon }d\beta_{i}+\frac{1}{2}{\hat{K}}_{t}\Xi \sigma_{b}{d\eta }_{t}\; \end{array}$$

where Ξ denotes the matrix Ξ(q), $\Xi_{Ci}$, i=1,2,3, denote the columns of the matrix Ξ, and the scalar processes $\beta_{i}$, and i=1,2,3 are the components of the vector Brownian motion $\beta_{t}$. Notice that Ξ is a function of the augmented process $\left\{{\hat{q}}_{t},{\tilde{q}}_{t}\right\}$, as it is a function of the quaternion q. Appending Equations (15) and (16) yields the following augmented SDE:

(17) $$\begin{array}{cc} \; & \left[\begin{array}{c} d{\hat{q}}_{t}\\ d{\tilde{q}}_{t} \end{array}\right]=\left[\begin{array}{c} \left(\frac{1}{2}\Omega_{t}-{\hat{K}}_{t}H_{t}\right){\hat{q}}_{t}\\ \left(\frac{1}{2}\Omega_{t}-{\hat{K}}_{t}H_{t}\right){\tilde{q}}_{t} \end{array}\right]dt+\sum_{i=1}^{3}\left[\begin{array}{c} O_{4\times 1}\\ -\frac{1}{2}\Xi_{Ci} \end{array}\right]\sigma_{\epsilon }d\beta_{i}+\left[\begin{array}{c} O_{4\times 3}\\ \frac{1}{2}{\hat{K}}_{t}\Xi \end{array}\right]\sigma_{b}{d\eta }_{t}\; \end{array}$$

where $O_{m\times n}$ denotes an m×n matrix of zeros. Equation (17) can be re-written in the following compact form:

(18) $$\begin{array}{c} dq_{t}^{a}=F^{a}q_{t}^{a}dt+\sum_{i=1}^{3}g_{2}^{i}\left(q_{t}^{a}\right)\sigma_{\epsilon }d\beta_{i}+G\left(q_{t}^{a}\right)\sigma_{b}{d\eta }_{t} \end{array}$$

where $F^{a}$, $g_{2}^{i}\left(q_{t}^{a}\right)$, and $G(q_{t}^{a})$ are effectively defined from Equation (17). Equation (17) provides a process equation for the augmented state $q_{t}^{a}$ with Brownian motions as the inputs, state-dependent input gain matrices, and here a state-linear drift term.

### 2.3. Hamilton-Jacobi-Bellman Inequality

The desired $L_{2}$-gain property will be satisfied if and only if the augmented system (18) is dissipative with respect to the supply rate $S\left(v\left(t\right),q_{t}^{a}\right)=\gamma^{2}{∥v\left(t\right)∥}^{2}-{∥{\tilde{q}}_{t}∥}^{2}$, for a given positive scalar γ [16]. Thus, a non-negative scalar-valued function, $V(q^{a},t)$, is sought that satisfies the fundamental property [17]:

(19) $$\begin{array}{cc} \; & E\left\{V\left(q_{t}^{a},t\right)\right\}\leq E\{V\left(q_{s}^{a},s\right)+\int_{s}^{t}\{\gamma^{2}\;∥v\left(\tau \right){∥}^{2}-∥{\tilde{q}}_{\tau }{∥}^{2}d\tau \}\;\;\;\;\forall \;0\leq s\leq t\leq T \end{array}$$

(20) $$\begin{array}{cc} \; & E\left\{V\left(q_{0}^{a},0\right)\right\}\leq \gamma^{2}\;E\{∥{\tilde{q}}_{0}{∥}^{2}\}\; \end{array}$$

for all $q^{a}$ and for all admissible v(t). When Equation (19) is satisfied, the function *V* is called a storage function for the supply rate *S*. A sufficient condition for Equation (19) is as follows:

(21) $$\begin{array}{c} E\left\{dV\left(q^{a},t\right)\right\}\leq E\{\gamma^{2}\;∥v\left(t\right){∥}^{2}-∥{\tilde{q}}_{t}{∥}^{2}\}\;\;\;\forall 0\leq t\leq T\; \end{array}$$

for all $q^{a}$ and for all admissible v(t), where dV is the Itô differential of the function *V*. Notice that the processes $\left\{\Omega_{t}\right\}$ and $\left\{H_{t}\right\}$ are observed and thus belong to the information pattern. Let $I^{t}$ denote the information pattern at time *t*, which includes the observations of the angular velocity and of the LOS until *t*, i.e., $I^{t}=\left\{{\left\{\omega \right\}}_{0}^{t},{\left\{b\right\}}_{0}^{t}\right\}$. In the development of the sufficiency conditions, one can use the following property of the expectation operator to write (21) as follows:

(22) $$\begin{array}{cc} \; & E\{E\{dV\left(q^{a},t\right)-\gamma^{2}∥v_{t}{∥}^{2}+∥{\tilde{q}}_{t}{∥}^{2}|I^{t}\}\}\leq 0\; \end{array}$$

for all $q^{a}$ and *t*. Then, a sufficient condition for (22) is

(23) $$\begin{array}{cc} \; & E\{dV\left(q^{a},t\right)-\gamma^{2}∥v_{t}{∥}^{2}+∥{\tilde{q}}_{t}{∥}^{2}|I^{t}\}\leq 0\; \end{array}$$

for all $q^{a}$ and *t*. Given the conditioning on $I^{t}$ in (23), the functions $\Omega_{t}$ and $H_{t}$ can be considered deterministic functions. Using the Itô differentiation rule ([19], p. 112) and dropping the expectation operator on both sides of Equation (21) yields the following sufficient condition, i.e., the Hamilton–Jacobi–Bellman (HJB) inequality for $V(q^{a},t)$:

(24) $$\begin{array}{c} \frac{\partial V}{\partial t}+\frac{\partial V}{\partial q^{a}}F^{a}q^{a}+\frac{1}{2}\sigma_{b}^{2}\mathrm{tr}\left\{GG^{T}\frac{\partial^{2}V}{\partial {q^{a}}^{2}}\right\}+\frac{1}{2}\sigma_{\epsilon }^{2}\sum_{i=1}^{3}{g_{2}^{i}}^{T}\frac{\partial^{2}V}{\partial {q^{a}}^{2}}g_{2}^{i}\leq \gamma^{2}{∥v∥}^{2}-{∥\tilde{q}∥}^{2}\; \end{array}$$

for all 0≤t≤T, $q^{a}$, and for all admissible v(t), where *G* and $g_{2}^{i}$ are functions of $q^{a}$. Dropping the integral and the expectation operators is a standard procedure in stochastic $H_{\infty }$ estimation and control [16] or in stochastic optimal control ([20], p. 321).

#### 2.3.1. Case Where $v=\{\sigma_{\epsilon },\sigma_{b}\}$

Assume both $\sigma_{\epsilon }$ and $\sigma_{b}$ are perturbations. Bringing all terms to the left-hand-side (LHS) of Equation (24) yields

(25) $$\begin{array}{c} \frac{\partial V}{\partial t}+\frac{\partial V}{\partial q^{a}}F^{a}q^{a}+{q^{a}}^{T}Lq^{a}+\left[\frac{1}{2}\mathrm{tr}\left\{GG^{T}\frac{\partial^{2}V}{\partial {q^{a}}^{2}}\right\}-\gamma^{2}\right]\sigma_{b}^{2}+\left[\frac{1}{2}\sum_{i=1}^{3}{g_{2}^{i}}^{T}\frac{\partial^{2}V}{\partial {q^{a}}^{2}}g_{2}^{i}-\gamma^{2}\right]\sigma_{\epsilon }^{2}\leq 0\; \end{array}$$

where *L* denotes the following 8×8 matrix

(26) $$\begin{array}{c} L=\left[\begin{array}{cc} O_{4} & O_{4}\\ O_{4} & I_{4} \end{array}\right]\; \end{array}$$

and $I_{4}$ denotes the four-dimensional identity matrix. For a solution to exist for all $q^{a}$ and for all admissible $\left\{\sigma_{\epsilon },\sigma_{b}\right\}$ the coefficients multiplying the arbitrary disturbance functions, $\sigma_{\epsilon }$ and $\sigma_{b}$, in Equation (25) must be negative, which yields the following conditions:

(27) $$\begin{array}{cc} \; & \frac{1}{2}\mathrm{tr}\left\{GG^{T}\frac{\partial^{2}V}{\partial {q^{a}}^{2}}\right\}-\gamma^{2}\leq 0 \end{array}$$

(28) $$\begin{array}{cc} \; & \frac{1}{2}\sum_{i=1}^{3}{g_{2}^{i}}^{T}\frac{\partial^{2}V}{\partial {q^{a}}^{2}}g_{2}^{i}-\gamma^{2}\leq 0\; \end{array}$$

for all 0≤t≤T, $q^{a}$. Thus, according to Equation (25), any non-zero disturbance will only add a negative term to the LHS, increasing the system’s dissipativity for the chosen supply rate. Henceforth, the worst-case scenario consists of vanishing disturbances, e.g.,

(29) $$\begin{array}{cc} \; & \sigma_{\epsilon }^{*}=0 \end{array}$$

(30) $$\begin{array}{cc} \; & \sigma_{b}^{*}=0\; \end{array}$$

Some interpretation of Equations (29) and (30) is required. It might appear at first sight counter-intuitive that the worst-case scenario for an estimator consists of vanishing noise variances. However, the proposed solution follows the attenuation $H_{\infty }$ criterion (12), not a minimum error condition. Henceforth, performance is measured via the ratio between the ($L_{2}$ norms of the) estimation error and the noise variance, not via the estimation error itself. It is thus not surprising that the proposed estimator will perform better, i.e., attenuate better in the presence of high variances than in the presence of low variances.

##### Summary

Using Equations (29) and (30) in Equation (25) yields the following sufficient conditions. For all $q^{a}$ and 0≤t≤T,

(31) $$\begin{array}{cc} \; & \frac{\partial V}{\partial t}+\frac{\partial V}{\partial q^{a}}F^{a}q^{a}+{q^{a}}^{T}Lq^{a}\leq 0 \end{array}$$

(32) $$\begin{array}{cc} \; & \frac{1}{2}\mathrm{tr}\left\{GG^{T}\frac{\partial^{2}V}{\partial {q^{a}}^{2}}\right\}-\gamma^{2}\leq 0 \end{array}$$

(33) $$\begin{array}{cc} \; & \frac{1}{2}\sum_{i=1}^{3}{g_{2}^{i}}^{T}\frac{\partial^{2}V}{\partial {q^{a}}^{2}}g_{2}^{i}-\gamma^{2}\leq 0\; \end{array}$$

#### 2.3.2. Case Where $v=\sigma_{\epsilon }$

Consider the case where $\sigma_{\epsilon }$ is the sole perturbation. In this case, the HJB inequality evaluated at the worst-case yields the following sufficient conditions: for all $q^{a}$ and 0≤t≤T,

(34) $$\begin{array}{cc} \; & \frac{\partial V}{\partial t}+\frac{\partial V}{\partial q^{a}}Fq^{a}+{q^{a}}^{T}Lq^{a}+\frac{1}{2}\sigma_{b}^{2}\mathrm{tr}\left\{GG^{T}\frac{\partial^{2}V}{\partial {q^{a}}^{2}}\right\}\leq 0 \end{array}$$

(35) $$\begin{array}{cc} \; & \frac{1}{2}\sum_{i=1}^{3}{g_{2}^{i}}^{T}\frac{\partial^{2}V}{\partial {q^{a}}^{2}}g_{2}^{i}-\gamma^{2}\leq 0\; \end{array}$$

In Equation (34), parameter $\sigma_{b}$ is a known deterministic parameter.

### 2.4. Storage Function V

A standard approach to circumvent the formidable task of solving the partial differential inequality for *V* [Equation (24)] consists of guessing the solution in a parameterized form and developing sufficient conditions for its parameters. The classical quadratic form for *V* will be used here:

(36) $$\begin{array}{c} V\left(q^{a},t\right)={q^{a}}^{T}P_{t}q^{a} \end{array}$$

where $P_{t}$ is assumed to be symmetric, positive, and block diagonal, i.e.,

(37) $$\begin{array}{c} P_{t}=\left[\begin{array}{cc} {\hat{P}}_{t} & O_{4}\\ O_{4} & {\tilde{P}}_{t} \end{array}\right] \end{array}$$

### 2.5. Sufficient Conditions on the Matrices $\hat{K}$, $\tilde{P}$, $\hat{P}$

Using Equations (36) and (37) in Equation (31), straightforward computations yield the following identity:

(38) $$\begin{array}{c} \frac{\partial V}{\partial t}+\frac{\partial V}{\partial q^{a}}F^{a}q^{a}+{q^{a}}^{T}Lq^{a}=\left[\begin{array}{cc} {{\hat{q}}_{t}}^{T} & {{\tilde{q}}_{t}}^{T} \end{array}\right]\left[\begin{array}{cc} \frac{d{\hat{P}}_{t}}{dt}+{\hat{F}}_{t}^{T}{\hat{P}}_{t}+{\hat{P}}_{t}{\hat{F}}_{t} & O_{4}\\ O_{4} & \frac{d{\tilde{P}}_{t}}{dt}+{\hat{F}}_{t}^{T}{\tilde{P}}_{t}+{\tilde{P}}_{t}{\hat{F}}_{t}+I_{4} \end{array}\right]\left[\begin{array}{c} {\hat{q}}_{t}\\ {\tilde{q}}_{t} \end{array}\right]\; \end{array}$$

where ${\hat{F}}_{t}$ is defined as follows:

(39) $$\begin{array}{c} {\hat{F}}_{t}=\frac{1}{2}\Omega_{t}-{\hat{K}}_{t}H_{t}\; \end{array}$$

Exploiting the following property of the matrix Ξ ([9], Eq. (8.20b)),

(40) $$\begin{array}{cc} \; & \Xi \Xi^{T}=q_{t}^{T}q_{t}I_{4}-q_{t}q_{t}^{T}\; \end{array}$$

yields the following identities

(41) $$\begin{array}{cc} \; & \frac{1}{2}\sum_{i=1}^{3}{g_{2}^{i}}^{T}\frac{\partial^{2}V}{\partial {q^{a}}^{2}}g_{2}^{i}=q_{t}^{T}\left[\frac{1}{4}\left(\mathrm{tr}{\tilde{P}}_{t}I_{4}-{\tilde{P}}_{t}\right)\right]q_{t} \end{array}$$

(42) $$\begin{array}{cc} \; & \frac{1}{2}\mathrm{tr}\left\{GG^{T}\frac{\partial^{2}V}{\partial {q^{a}}^{2}}\right\}=q_{t}^{T}\left\{\frac{1}{4}\left[\mathrm{tr}\left({\hat{K}}_{t}^{T}{\tilde{P}}_{t}{\hat{K}}_{t}\right)I_{4}-{\hat{K}}_{t}^{T}{\tilde{P}}_{t}{\hat{K}}_{t}\right]\right\}q_{t}\; \end{array}$$

#### 2.5.1. Convexity Condition with Respect to $\sigma_{\epsilon }$

Using Equation (41) in Equation (33) [Equation (35)], and noting that the inequality must be verified for all $q_{t}$ yields the following condition on ${\tilde{P}}_{t}$:

(43) $$\begin{array}{c} \frac{1}{4}\left(\mathrm{tr}{\tilde{P}}_{t}I_{4}-{\tilde{P}}_{t}\right)-\gamma^{2}I_{4}\leq 0 \end{array}$$

This condition must be satisfied in both cases, whether $\sigma_{b}$ is known or is an unknown perturbation. The latter condition can be easily reformulated in terms of the characteristic values of the symmetric positive matrix $\tilde{P}$. Let ${\tilde{\lambda }}_{i}$, i=1,2,3,4, denote the four positive eigenvalues of $\tilde{P}$, then Equation (43) is equivalent to the following condition:

(44) $$\begin{array}{cc} \; & \frac{1}{4}\max\left\{{\tilde{\lambda }}_{2}+{\tilde{\lambda }}_{3}+{\tilde{\lambda }}_{4},{\tilde{\lambda }}_{1}+{\tilde{\lambda }}_{3}+{\tilde{\lambda }}_{4},{\tilde{\lambda }}_{1}+{\tilde{\lambda }}_{2}+{\tilde{\lambda }}_{4},{\tilde{\lambda }}_{1}+{\tilde{\lambda }}_{2}+{\tilde{\lambda }}_{3}\right\}\leq \gamma^{2}\; \end{array}$$

#### 2.5.2. Case Where $v=\{\sigma_{\epsilon },\sigma_{b}\}$

Using Equation (42) in Equation (32), and noting that the inequality must be verified for all $q_{t}$, yields the following condition on ${\tilde{P}}_{t}$ and $\hat{K}$:

(45) $$\begin{array}{cc} \; & \frac{1}{4}\left[\mathrm{tr}\left({\hat{K}}_{t}^{T}{\tilde{P}}_{t}{\hat{K}}_{t}\right)I_{4}-{\hat{K}}_{t}^{T}{\tilde{P}}_{t}{\hat{K}}_{t}\right]-\gamma^{2}I_{4}\leq 0\; \end{array}$$

Using Equation (38) in Equation (31) yields two uncoupled differential matrix inequalities for ${\hat{P}}_{t}$ and ${\tilde{P}}_{t}$, respectively:

(46) $$\begin{array}{cc} \; & \frac{d{\hat{P}}_{t}}{dt}+F_{t}^{T}{\hat{P}}_{t}+{\hat{P}}_{t}F_{t}\leq 0 \end{array}$$

(47) $$\begin{array}{cc} \; & \frac{d{\tilde{P}}_{t}}{dt}+F_{t}^{T}{\tilde{P}}_{t}+{\tilde{P}}_{t}F_{t}+I_{4}\leq 0\; \end{array}$$

Assuming that the matrices ${\hat{P}}_{t}$ and ${\tilde{P}}_{t}$ are identical for the sake of simplicity allows dropping Equation (46). Combining Equations (43), (45) and (47) yields the inequalities to be solved for $\hat{K}$ and $\tilde{P}$.

(48) $$\begin{array}{cc} \; & \frac{d{\tilde{P}}_{t}}{dt}+F_{t}^{T}{\tilde{P}}_{t}+{\tilde{P}}_{t}F_{t}+I_{4}\leq 0 \end{array}$$

(49) $$\begin{array}{cc} \; & \frac{1}{4}\left(\mathrm{tr}{\tilde{P}}_{t}I_{4}-{\tilde{P}}_{t}\right)-\gamma^{2}I_{4}\leq 0 \end{array}$$

(50) $$\begin{array}{cc} \; & \frac{1}{4}\left(\mathrm{tr}M_{t}I_{4}-M_{t}\right)-\gamma^{2}I_{4}\leq 0\; \end{array}$$

where

(51) $$\begin{array}{cc} \; & F_{t}=\frac{1}{2}\Omega_{t}-\hat{K}H_{t} \end{array}$$

(52) $$\begin{array}{cc} \; & M_{t}={\hat{K}}^{T}{\tilde{P}}_{t}\hat{K}\; \end{array}$$

Notice that the left-hand sides (LHS) of the above inequalities are independent of the quaternion estimate; thus, the filter gain $\hat{K}$ is also independent of ${\hat{q}}_{t}$. It will be denoted by $K_{t}$ in the following.

##### Sufficient Conditions in the Form of Linear Matrix Inequalities

As the above inequalities are not linear with respect to $\tilde{P}$ and *K*, some manipulations are required in order to bring them to a Linear Matrix Inequality (LMI) structure. The bilinear dependence with respect to $\tilde{P}$ and *K* is readily coped with via a standard parametrization approach. Let ${\tilde{Y}}_{t}$ denote the following four-dimensional matrix:

(53) $$\begin{array}{c} {\tilde{Y}}_{t}={\tilde{P}}_{t}K_{t}\; \end{array}$$

then, using Equation (53) in Equation (48) yields

(54) $$\begin{array}{c} \frac{d{\tilde{P}}_{t}}{dt}+\frac{1}{2}\left(\Omega_{t}^{T}{\tilde{P}}_{t}+{\tilde{P}}_{t}\Omega_{t}\right)-\left(H_{t}^{T}{\tilde{Y}}_{t}^{T}+{\tilde{Y}}_{t}H_{t}\right)+I_{4}\leq 0\; \end{array}$$

To circumvent the difficulty arising from the quadratic structure of $M_{t}$ with respect to ${\tilde{P}}_{t}$ and *K*, a symmetric positive definite matrix $W_{t}$ is sought, such that

(55) $$\begin{array}{c} M_{t}-W_{t}={\tilde{Y}}_{t}^{T}{\tilde{P}}_{t}^{-1}{\tilde{Y}}_{t}-W_{t}\leq 0 \end{array}$$

Notice that ${\tilde{P}}_{t}^{-1}$ exists as ${\tilde{P}}_{t}$ is assumed to be positive definite. Then, the following bounds on the LHS of Equation (50) are used:

(56) $$\begin{array}{c} \frac{1}{4}\left(\mathrm{tr}M_{t}I_{4}-M_{t}\right)-\gamma^{2}I_{4}\leq \left(\frac{1}{4}\mathrm{tr}M_{t}-\gamma^{2}\right)I_{4}\leq \left(\frac{1}{4}\mathrm{tr}W_{t}-\gamma^{2}\right)I_{4}\leq 0\; \end{array}$$

and Equation (50) is replaced with the following sufficient condition on *W*:

(57) $$\begin{array}{cc} \; & \frac{1}{4}\mathrm{tr}W_{t}-\gamma^{2}\leq 0\; \end{array}$$

where *W*, $\tilde{Y}$, and $\tilde{P}$ satisfy Equation (55), which by the Schur complement can be written as the following LMI:

(58) $$\begin{array}{cc} \; & \left[\begin{array}{cc} -W_{t} & -{\tilde{Y}}_{t}\\ -{\tilde{Y}}_{t}^{T} & -{\tilde{P}}_{t} \end{array}\right]\leq 0\; \end{array}$$

Notice that the successive bounds in Equation (56) yield a sufficient condition for the attenuation filtering problem.

#### 2.5.3. Case Where $v=\sigma_{\epsilon }$

Using Equation (42) and the definition of $q^{a}$ yields the following identity:

(59) $$\begin{array}{cc} \frac{1}{2}\sigma_{b}^{2}\mathrm{tr}\left\{GG^{T}\frac{\partial^{2}V}{\partial {q^{a}}^{2}}\right\}\; & =q_{t}^{T}\left\{\frac{\sigma_{b}^{2}}{4}\left[\begin{array}{c} \mathrm{tr}\left({\hat{K}}_{t}^{T}{\tilde{P}}_{t}{\hat{K}}_{t}\right)I_{4}-{\hat{K}}_{t}^{T}{\tilde{P}}_{t}{\hat{K}}_{t} \end{array}\right]\right\}q_{t}\\ \; & =\left[\begin{array}{cc} {{\hat{q}}_{t}}^{T} & {{\tilde{q}}_{t}}^{T} \end{array}\right]\left[\begin{array}{c} I_{4}\\ I_{4} \end{array}\right]\left\{\frac{\sigma_{b}^{2}}{4}\left[\begin{array}{c} \mathrm{tr}\left({\hat{K}}_{t}^{T}{\tilde{P}}_{t}{\hat{K}}_{t}\right)I_{4}-{\hat{K}}_{t}^{T}{\tilde{P}}_{t}{\hat{K}}_{t} \end{array}\right]\right\}\left[\begin{array}{cc} I_{4} & I_{4} \end{array}\right]\left[\begin{array}{c} {\hat{q}}_{t}\\ {\tilde{q}}_{t} \end{array}\right]\; \end{array}$$

Using Equations (38) and (59) in Equation (34) yields

(60) $$\begin{array}{c} \left[\begin{array}{cc} {\hat{q}}_{t}^{T} & {{\tilde{q}}_{t}}^{T} \end{array}\right]\left[\begin{array}{cc} \frac{d{\hat{P}}_{t}}{dt}+{\hat{F}}_{t}^{T}{\hat{P}}_{t}+{\hat{P}}_{t}{\hat{F}}_{t}+\frac{\sigma_{b}^{2}}{4}\left[\begin{array}{c} \mathrm{tr}{\hat{M}}_{t}I_{4}-{\hat{M}}_{t} \end{array}\right]\; & \frac{\sigma_{b}^{2}}{4}\left[\begin{array}{c} \mathrm{tr}{\hat{M}}_{t}I_{4}-{\hat{M}}_{t} \end{array}\right]\\ \frac{\sigma_{b}^{2}}{4}\left[\begin{array}{c} \mathrm{tr}{\hat{M}}_{t}I_{4}-{\hat{M}}_{t} \end{array}\right]\; & \frac{d{\tilde{P}}_{t}}{dt}+{\hat{F}}_{t}^{T}{\tilde{P}}_{t}+{\tilde{P}}_{t}{\hat{F}}_{t}+\frac{\sigma_{b}^{2}}{4}\left[\begin{array}{c} \mathrm{tr}{\hat{M}}_{t}I_{4}-{\hat{M}}_{t} \end{array}\right]+I_{4} \end{array}\right]\left[\begin{array}{c} {\hat{q}}_{t}\\ {\tilde{q}}_{t} \end{array}\right]\leq 0\; \end{array}$$

for all $\left({\hat{q}}_{t},{\tilde{q}}_{t},t\right)$, where ${\hat{M}}_{t}={\hat{K}}_{t}^{T}{\tilde{P}}_{t}{\hat{K}}_{t}$. Assuming that $\hat{P}=\tilde{P}$ as in the previous case yields the following matrix differential inequality

(61) $$\begin{array}{c} \left[\begin{array}{cc} \frac{d{\tilde{P}}_{t}}{dt}+F^{T}{\tilde{P}}_{t}+{\tilde{P}}_{t}F+\frac{\sigma_{b}^{2}}{4}\left[\begin{array}{c} \left(\mathrm{tr}M\right)I_{4}-M \end{array}\right]\; & \frac{\sigma_{b}^{2}}{4}\left[\begin{array}{c} \left(\mathrm{tr}M\right)I_{4}-M \end{array}\right]\\ \frac{\sigma_{b}^{2}}{4}\left[\begin{array}{c} \left(\mathrm{tr}M\right)I_{4}-M \end{array}\right]\; & \frac{d{\tilde{P}}_{t}}{dt}+F^{T}{\tilde{P}}_{t}+{\tilde{P}}_{t}F+I_{4}+\frac{\sigma_{b}^{2}}{4}\left[\begin{array}{c} \left(\mathrm{tr}M\right)I_{4}-M \end{array}\right] \end{array}\right]\leq 0\; \end{array}$$

for all 0≤t≤T, where

(62) $$\begin{array}{cc} \; & F=\frac{1}{2}\Omega_{t}-K_{t}H_{t} \end{array}$$

(63) $$\begin{array}{cc} \; & M=K_{t}^{T}{\tilde{P}}_{t}K_{t} \end{array}$$

Thus, when $\sigma_{b}$ is a known parameter, Equations (61)–(63) and Equation (43) are the sufficient conditions for $\tilde{P}$ and *K*.

##### Sufficient Conditions in the Form of LMI

Introducing a matrix variable $W_{t}$ that satisfies Equations (53) and (55), and using the same upper bounds sequence as in Equation (56) yields the following differential LMI for this case:

(64) $$\begin{array}{cc} \; & \left[\begin{array}{cc} \frac{d{\tilde{P}}_{t}}{dt}+\frac{1}{2}\left(\Omega_{t}^{T}{\tilde{P}}_{t}+{\tilde{P}}_{t}\Omega_{t}\right)-\left(H_{t}^{T}{\tilde{Y}}_{t}^{T}+{\tilde{Y}}_{t}H_{t}\right)+\frac{\sigma_{b}^{2}}{4}\left(\mathrm{tr}W_{t}\right)I_{4}\; & \;\frac{\sigma_{b}^{2}}{4}\left(\mathrm{tr}W_{t}\right)I_{4}\\ \;\frac{\sigma_{b}^{2}}{4}\left(\mathrm{tr}W_{t}\right)I_{4}\; & \;\frac{d{\tilde{P}}_{t}}{dt}+\frac{1}{2}\left(\Omega_{t}^{T}{\tilde{P}}_{t}+{\tilde{P}}_{t}\Omega_{t}\right)-\left(H_{t}^{T}{\tilde{Y}}_{t}^{T}+{\tilde{Y}}_{t}H_{t}\right)+I_{4}+\frac{\sigma_{b}^{2}}{4}\left(\mathrm{tr}W_{t}\right)I_{4} \end{array}\right]\leq 0\; \end{array}$$

### 2.6. Quaternion Stochastic $H_{\infty }$ Filters Summary

Given ${\hat{q}}_{0}$, choose $\tilde{P}\left(0\right)$, such that Equation (20) is satisfied. Solve the following set of (differential) LMIs for ${\tilde{P}}_{t}={\tilde{P}}_{t}^{T}>0\in R^{4}$, ${\tilde{Y}}_{t}\in R^{4}$, and $W_{t}=W_{t}^{T}>0\in R^{4}$:

#### 2.6.1. Case Where $v=\{\sigma_{\epsilon },\sigma_{b}\}$


(65) $$\begin{array}{cc} \; & \frac{d{\tilde{P}}_{t}}{dt}+\frac{1}{2}\left(\Omega_{t}^{T}{\tilde{P}}_{t}+{\tilde{P}}_{t}\Omega_{t}\right)-\left(H_{t}^{T}{\tilde{Y}}_{t}^{T}+{\tilde{Y}}_{t}H_{t}\right)+I_{4}\leq 0 \end{array}$$

(66) $$\begin{array}{cc} \; & \left[\begin{array}{cc} -W_{t} & -{\tilde{Y}}_{t}\\ -{\tilde{Y}}_{t}^{T} & -{\tilde{P}}_{t} \end{array}\right]\leq 0 \end{array}$$

(67) $$\begin{array}{cc} \; & \frac{1}{4}\left(\mathrm{tr}{\tilde{P}}_{t}I_{4}-{\tilde{P}}_{t}\right)-\gamma^{2}I_{4}\leq 0 \end{array}$$

(68) $$\begin{array}{cc} \; & \frac{1}{4}\mathrm{tr}W_{t}-\gamma^{2}\leq 0\; \end{array}$$

#### 2.6.2. Case Where $v=\sigma_{\epsilon }$


(69) $$\begin{array}{cc} \; & \left[\begin{array}{cc} \frac{d{\tilde{P}}_{t}}{dt}+\frac{1}{2}\left(\Omega_{t}^{T}{\tilde{P}}_{t}+{\tilde{P}}_{t}\Omega_{t}\right)-\left(H_{t}^{T}{\tilde{Y}}_{t}^{T}+{\tilde{Y}}_{t}H_{t}\right)+\frac{\sigma_{b}^{2}}{4}\left(\mathrm{tr}W_{t}\right)I_{4}\; & \;\frac{\sigma_{b}^{2}}{4}\left(\mathrm{tr}W_{t}\right)I_{4}\\ \;\frac{\sigma_{b}^{2}}{4}\left(\mathrm{tr}W_{t}\right)I_{4}\; & \;\frac{d{\tilde{P}}_{t}}{dt}+\frac{1}{2}\left(\Omega_{t}^{T}{\tilde{P}}_{t}+{\tilde{P}}_{t}\Omega_{t}\right)-\left(H_{t}^{T}{\tilde{Y}}_{t}^{T}+{\tilde{Y}}_{t}H_{t}\right)+I_{4}+\frac{\sigma_{b}^{2}}{4}\left(\mathrm{tr}W_{t}\right)I_{4} \end{array}\right]\leq 0 \end{array}$$

(70) $$\begin{array}{cc} \; & \left[\begin{array}{cc} -W_{t} & -{\tilde{Y}}_{t}\\ -{\tilde{Y}}_{t}^{T} & -{\tilde{P}}_{t} \end{array}\right]\leq 0 \end{array}$$

(71) $$\begin{array}{cc} \; & \frac{1}{4}\left(\mathrm{tr}{\tilde{P}}_{t}I_{4}-{\tilde{P}}_{t}\right)-\gamma^{2}I_{4}\leq 0\; \end{array}$$

For any pair of matrices $\left({\tilde{Y}}_{t},{\tilde{P}}_{t}\right)$, compute the gain $K_{t}$ using

(72) $$\begin{array}{cc} \; & K_{t}={\tilde{P}}_{t}^{-1}{\tilde{Y}}_{t}\; \end{array}$$

and compute the estimated quaternion via the estimator differential equation

(73) $$\begin{array}{cc} \; & {\dot{\hat{q}}}_{t}=\left[\frac{1}{2}\Omega_{t}-K_{t}H_{t}\right]{\hat{q}}_{t}\; \end{array}$$

**Remark** **1.** *The estimator in Equation (73) is not designed to preserve the estimate quaternion unit-norm property. For that purpose, a normalization stage of the estimate is performed along the estimation process [4,7]*

(74) $$\begin{array}{cc} \; & \hat{q}=\frac{\hat{q}}{∥\hat{q}∥}\; \end{array}$$

**Remark** **2.** *A key feature of the above filters lies in the fact that the gain computations are independent of the estimated process. As a result, the gain values are insensitive to the initial estimation errors, which are often causes of divergence in linearization-based filtering techniques, like the extended Kalman filter. An additional essential by-product is that the estimate differential equation (73) can be integrated as an ordinary differential equation.*

**Remark** **3.** *The omission of the damping term in Equation (2) does not affect the estimator’s stability as Equation (73) remains valid. Yet, it affects the gain calculations due to additional terms in the LMI governing the sufficient condition related to $\sigma_{\epsilon }$. Neglecting this impact is justified in our approach because the magnitude of the term $\frac{3}{4}\sigma_{\epsilon }^{2}$ is typically extremely small and because the drift of the length of the true quaternion is a valid assumption in the $H_{\infty }$ estimation framework.*

**Remark** **4.** *The above algorithms are solved using standard primal-dual interior-point methods, as implemented in SeDuMi [21,22]. The method formulates a minimization problem over γ subject to the constraints described in Equations (65)–(68). For the solver SeDuMi, an assessment of the computational complexity is $O(n^{4})$ for the $2n^{2}+n$ decision variables, where n=4. Compared with the standard computational complexity of a Kalman filter, i.e., $O(n^{3})$, this yields a ratio of 4.*

**Remark** **5.** *Inspired by [23], discrete approximations of the differential LMIs are developed via finite-difference formulas. For example, Equation (65) is implemented as follows:*

(75) $$\begin{array}{c} \frac{{\tilde{P}}_{k+1}-{\tilde{P}}_{k}}{\Delta t}+\frac{1}{2}\left(\Omega_{k+1}^{T}{\tilde{P}}_{k+1}+{\tilde{P}}_{k+1}\Omega_{k+1}\right)-\left(H_{k+1}^{T}{\tilde{Y}}_{k+1}^{T}+{\tilde{Y}}_{k+1}H_{k+1}\right)+I_{4}\leq 0\; \end{array}$$

*where Δt denotes the time increment, and k=0,1,…,N=T/Δt.*

## 3. Quaternion and Gyro Drift Estimation

### 3.1. Statement of the Problem

Assuming that the rate gyro error consists of white noise and a bias, we consider the following stochastic dynamical system in Itô form:

(76) $$\begin{array}{cc} \; & dq_{t}=\frac{1}{2}\Omega \left(\omega_{t}-c_{t}\right)q_{t}dt-\frac{1}{2}\Xi \left(q_{t}\right)\sigma_{\epsilon }\left(t\right){d\beta }_{t};\;q\left(0\right)\overset{a.e.}{=}q_{0};\;t\in \left[0,T\right] \end{array}$$

(77) $$\begin{array}{cc} \; & dc_{t}=\sigma_{c}\left(t\right)d\nu_{t};\;c\left(0\right)\overset{a.e.}{=}c_{0} \end{array}$$

(78) $$\begin{array}{cc} \; & dy_{t}=H_{t}q_{t}dt-\frac{1}{2}\Xi \left(q_{t}\right)\sigma_{b}\left(t\right){d\eta }_{t}\; \end{array}$$

where $c_{t}$ denotes the additive drift, modeled as a random walk process with mean $c_{0}$ and variance parameter $\sigma_{c}\left(t\right)$. In Equation (77), $\nu_{t}$ denotes a standard Brownian motion that is independent of $\beta_{t}$ and $\eta_{t}$. Equations (76) and (77) stem from a straightforward extension of the quaternion SDE of Section 2.

The filtering problem consists of estimating the quaternion $q_{t}$ and the gyro drift $c_{t}$ from the LOS measurements in the presence of unknown noise standard deviations $\sigma_{\epsilon }\left(t\right)$, $\sigma_{b}\left(t\right)$, and $\sigma_{c}\left(t\right)$. Assuming that $\sigma_{\epsilon }\left(t\right)$, $\sigma_{b}\left(t\right)$, and $\sigma_{c}\left(t\right)$ are stochastic non-anticipative processes with finite second-order moments, we consider the following estimator:

(79) $$\begin{array}{cc} \; & d{\hat{q}}_{t}=\frac{1}{2}\Omega \left(\omega_{t}-{\hat{c}}_{t}\right){\hat{q}}_{t}dt+K_{q}\left(dy_{t}-{\hat{q}}_{t}dt\right) \end{array}$$

(80) $$\begin{array}{cc} \; & d{\hat{c}}_{t}=K_{c}\left(dy_{t}-{\hat{q}}_{t}dt\right) \end{array}$$

(81) $$\begin{array}{cc} \; & \hat{q}\left(0\right)={\hat{q}}_{0},\hat{c}\left(0\right)={\hat{c}}_{0}\; \end{array}$$

Let ${\tilde{q}}_{t}$ and ${\tilde{c}}_{t}$ denote the additive quaternion and biases estimation error, i.e.,

(82) $$\begin{array}{cc} \; & {\tilde{q}}_{t}=q_{t}-{\hat{q}}_{t} \end{array}$$

(83) $$\begin{array}{cc} \; & {\tilde{c}}_{t}=c_{t}-{\hat{c}}_{t}\; \end{array}$$

Given a scalar γ>0, we seek the gains $K_{q},K_{c}$, such that the following $H_{\infty }$ criterion is satisfied:

(84) $$\begin{array}{c} E\{\int_{0}^{T}(∥{\tilde{q}}_{t}{∥}^{2}+∥{\tilde{c}}_{t}{∥}^{2})dt\}\leq \gamma^{2}E\{∥{\tilde{q}}_{0}{∥}^{2}+∥{\tilde{c}}_{0}{∥}^{2}+\int_{0}^{T}∥v_{t}{∥}^{2}dt\} \end{array}$$

under the constraints (76)–(78), and where $v_{t}$ denotes the augmented process of admissible disturbance functions, i.e., $v_{t}=\left\{\sigma_{\epsilon }\left(t\right),\sigma_{b}\left(t\right),\sigma_{c}\left(t\right)\right\}$. Whenever Equation (84) is true, it is said that the $L_{2}$-gain property is satisfied from $\left\{{\tilde{q}}_{0},{\tilde{c}}_{0},v_{t}\right\}$ to $\left\{{\tilde{q}}_{t},{\tilde{c}}_{t}\right\}$, for 0≤t≤T.

### 3.2. Design Model Development

The SDE of the quaternion-drift system is compactly rewritten as follows:

(85) $$\begin{array}{cc} \; & \left[\begin{array}{c} dq_{t}\\ dc_{t} \end{array}\right]=\left[\begin{array}{c} \frac{1}{2}\Omega \left(\omega_{t}-c_{t}\right)q_{t}\\ O_{3\times 1} \end{array}\right]dt+\left[\begin{array}{cc} -\frac{1}{2}\Xi \left(q_{t}\right) & O_{4\times 3}\\ O_{3} & I_{3}\; \end{array}\right]\left[\begin{array}{cc} \sigma_{\epsilon }\left(t\right)I_{3} & O_{3}\\ O_{3} & \sigma_{c}\left(t\right)I_{3} \end{array}\right]\left[\begin{array}{c} {d\beta }_{t}\\ d\nu_{t} \end{array}\right] \end{array}$$

and the estimator is rewritten as follows:

(86) $$\begin{array}{cc} \; & \left[\begin{array}{c} d{\hat{q}}_{t}\\ d{\hat{c}}_{t} \end{array}\right]=\left[\begin{array}{c} \left[\frac{1}{2}\Omega \left(\omega_{t}-{\hat{c}}_{t}\right)-K_{q}H_{t}\right]\\ -K_{c}H_{t} \end{array}\right]{\hat{q}}_{t}dt\\ \; & {\hat{q}}_{t}\left(0\right)={\hat{q}}_{0};\;{\hat{c}}_{t}\left(0\right)={\hat{c}}_{0}\; \end{array}$$

The augmented process $\left\{{\hat{q}}_{t},{\hat{c}}_{t},\tilde{q},\tilde{c}\right\}$ is governed by the following SDE

(87) $$\begin{array}{cc} \left[\begin{array}{c} d{\hat{q}}_{t}\\ d{\hat{c}}_{t}\\ d{\tilde{q}}_{t}\\ d{\tilde{c}}_{t} \end{array}\right] & =\left[\begin{array}{cccc} \frac{1}{2}\Omega_{t}-K_{q}H_{t} & -\frac{1}{2}\Xi \left({\hat{q}}_{t}\right) & O & O\\ -K_{c}H_{t} & O & O & O\\ O & O & \frac{1}{2}\Omega_{t}-K_{q}H_{t} & -\frac{1}{2}\Xi \left({\hat{q}}_{t}\right)\\ O & O & -K_{c}H_{t} & O \end{array}\right]\left[\begin{array}{c} {\hat{q}}_{t}\\ {\hat{c}}_{t}\\ {\tilde{q}}_{t}\\ {\tilde{c}}_{t} \end{array}\right]dt\\ & +\left[\begin{array}{c} O_{4\times 3}\\ O_{3}\\ -\frac{1}{2}\Xi \left({\hat{q}}_{t}\right)\\ O_{3} \end{array}\right]\sigma_{\epsilon }\left(t\right){d\beta }_{t}+\left[\begin{array}{c} O_{4\times 3}\\ O_{3}\\ O_{4\times 3}\\ I_{3} \end{array}\right]\sigma_{c}\left(t\right)d\nu_{t}+\left[\begin{array}{c} O_{4\times 3}\\ O_{4\times 3}\\ K_{q}\frac{1}{2}\Xi \left({\hat{q}}_{t}\right)\\ K_{c}\frac{1}{2}\Xi \left({\hat{q}}_{t}\right) \end{array}\right]\sigma_{b}\left(t\right){d\eta }_{t}\; \end{array}$$

where second-order terms with respect to the noises $\beta_{t}$, $\nu_{t}$, and $\eta_{t}$ and to the estimation errors $\tilde{q}$, $\tilde{c}$ have been neglected. Equation (87) may be re-written in the following compact form:

(88) $$\begin{array}{cc} \; & dq_{t}^{a}=F^{a}q_{t}^{a}dt+G_{1}\left(q_{t}^{a}\right)\sigma_{\epsilon }\left(t\right){d\beta }_{t}+G_{2}\left(q_{t}^{a}\right)\sigma_{c}\left(t\right)d\nu_{t}+G\left(q_{t}^{a}\right)\sigma_{b}\left(t\right){d\eta }_{t}\; \end{array}$$

The remainder of the filter development is straightforward and is omitted for the sake of brevity.

## 4. Numerical Simulation

This section is concerned with the numerical validation of the proposed approach in the drift-free case.

### 4.1. Description

Consider a spacecraft rotating around its center of mass with the following time-varying inertial angular velocity vector, $\omega^{o}\left(t\right)$:

(89) $$\begin{array}{cc} \; & \omega^{o}\left(t\right)={\left[1,\;-1,\;1\right]}^{T}\sin\left(2\pi t/150\right)\;\left[\deg/s\right]\; \end{array}$$

The measured angular velocity is computed according to

(90) $$\begin{array}{cc} \; & \omega \left(t\right)=\omega^{o}\left(t\right)+\sigma_{\epsilon }\epsilon \left(t\right)\; \end{array}$$

where ϵ(t) is a standard zero-mean white Gaussian noise, e.g., $E\left\{\epsilon \left(t\right)\epsilon {\left(\tau \right)}^{T}\right\}=I_{3}\delta \left(t-\tau \right)$. Typical values of low-grade gyros are used, i.e., $\sigma_{\epsilon }\in \left[10^{-4},10^{0}\right]$ [rad/$\sqrt{s}$]. A time-varying line-of-sight measurement, $b_{t}$, is assumed to be acquired. It is computed via the classical vector measurement model:

(91) $$\begin{array}{cc} \; & b_{t}=A\left(q_{t}\right)r_{t}+\sigma_{b}{\delta b}_{t}\; \end{array}$$

where r(t) is randomly generated using a zero-mean standard multivariate normal distribution and the attitude matrix $A(q_{t})$ is expressed as follows:

(92) $$\begin{array}{cc} \; & A\left(q_{t}\right)=\left(q_{t}^{2}-e_{t}^{T}e_{t}\right)I_{3}+2e_{t}e_{t}^{T}-2q\left[e_{t}\times \right]\; \end{array}$$

As $b_{t}$ and $r_{t}$ are unit norm vectors the error ${\delta b}_{t}$ is a zero-mean white Gaussian noise with $E\left\{{\delta b}_{t}{\delta b}_{\tau }^{T}\right\}≃\left(I_{3}-b_{t}b_{t}^{T}\right)\delta \left(t-\tau \right)$. Typical values of coarse and fine attitude sensors are used, i.e., $\sigma_{b}\in \left[10^{-5},10^{-1}\right]$[rad]. The true initial quaternion is $q\left(0\right)={\left[1,1,1,1\right]}^{T}/\sqrt{4}$. The filter is initialized with $\hat{q}\left(0\right)=\left[0,0,0,1\right]$ and $\tilde{P}\left(0\right)=10I_{4}$, unless stated otherwise. Monte-Carlo simulations (50 runs) are run over time spans varying from 500 s, i.e., several periods of the angular velocity variations, to 6000 s, i.e., approximately one revolution of a low Earth orbit satellite. The simulation sampling time Δt is set to 0.1 s for both the gyro and the attitude sensing. The novel stochastic $H_{\infty }$ quaternion filter (QHF), a typical multiplicative EKF (MEKF [3]), an unscented quaternion filter (UQF [5]), and the quaternion Kalman filter introduced in [7] (QKF) are implemented for comparison.

### 4.2. Attenuation of Gyro $\sigma_{\epsilon }$

In this section, the numerical study focuses on the impact of the gyro perturbation $\sigma_{\epsilon }$ on the attenuation performance of the QHF. For this purpose, the parameter $\sigma_{\epsilon }$ is set to various known values, while $\sigma_{b}$ is kept equal to $10^{-6}$ radian. Table 1 presents the values of Monte-Carlo (MC) averages over 500 runs lasting 500 s each of $\delta_{q}=\max_{t}∥q_{t}-{\hat{q}}_{t}∥$ and of the ratio $\frac{\delta q}{\sigma_{\epsilon }\sqrt{\Delta t}}$, where Δt=0.1 s is the gyro sampling time. The former is a measure of attitude estimation accuracy, while the latter is a measure of attenuation performance. It can be seen that the QHF always converges, that the estimation accuracy is satisfying despite the extreme values of $\sigma_{\epsilon }$, albeit degraded as $\sigma_{\epsilon }$ increases, and that the attenuation performance improves with $\sigma_{\epsilon }$.

Additional MC simulations (500 runs) were performed while varying the parameters $\sigma_{\epsilon }$ and $\sigma_{b}$. Table 2 depicts the ratios of the time averages (over 6000 s) of the angular error, δϕ, of the QHF over the QKF. The error δϕ is extracted from the rotational quaternion error’s fourth component. The magnitude of δϕ in the QHF appears in parenthesis (in degree) above the ratios. For a given $\sigma_{\epsilon }$, the values of δϕ and the ratios increase with $\sigma_{b}$ because the attenuation quality is impaired. It turns out that the ratios are smaller than one in almost all test cases, i.e., the QHF produces a smaller bias than the QKF. The above results suggest that the QHF is advantageous when using low-grade gyros (high $\sigma_{\epsilon }$) with fine LOS sensors (low $\sigma_{b}$).

### 4.3. Attenuation of $\sigma_{\epsilon }$ and $\sigma_{b}$

Next, we test the performances of the QHF when both $\sigma_{\epsilon }$ and $\sigma_{b}$ are unknown. For this purpose, we evaluate the actual attenuation ratio AR(T), which is defined as follows:

(93) $$\begin{array}{cc} \; & AR\left(T\right)=\frac{E\{\int_{0}^{T}∥\tilde{q}\left(t\right){∥}^{2}dt\}}{E\{∥\tilde{q}\left(0\right){∥}^{2}+\int_{0}^{T}\left(\sigma_{\epsilon }^{2}+\sigma_{b}^{2}\right)dt\}}\; \end{array}$$

where the final time *T* is 500 s, the integrals are numerically computed using a time step Δt=0.1 s, and the expectations are computed as MC averages over 500 runs. Table 3 shows the values of AR(500) for various $\sigma_{\epsilon }$ and $\sigma_{b}$. It also features the steady-state MC means of the best-guaranteed level of attenuation, $\gamma_{QHF}^{2}$, which is calculated within the QHF.

**Figure 1:**
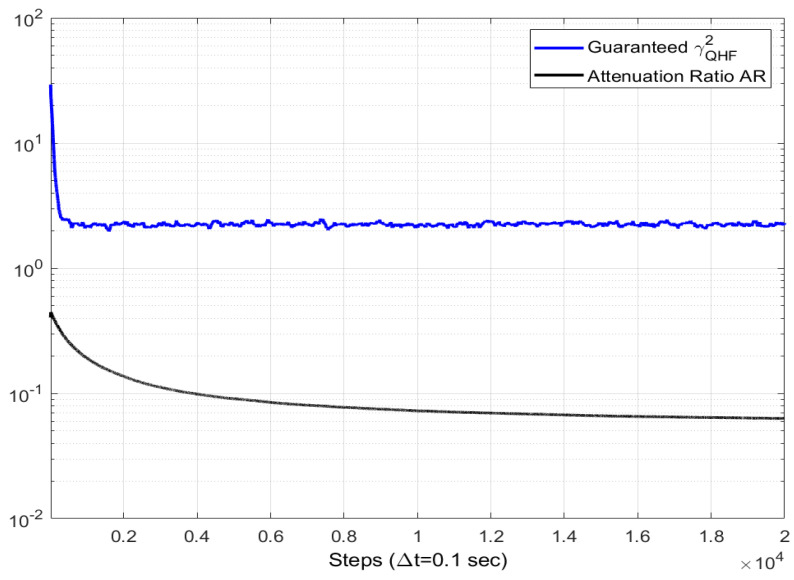
Time histories of the Attenuation Ratio (black line) and the best guaranteed bound $\gamma_{QHF}^{2}$ (blue line). 500 MC runs. $\left(\sigma_{\epsilon },\sigma_{b}\right)=\left(0.1,0.1\right)$.

**Figure 2:**
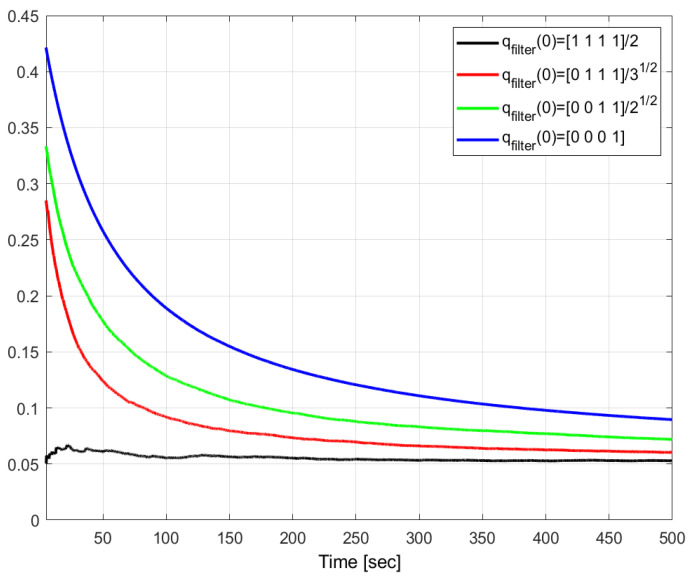
Time histories of the MC-mean of the Attenuation Ratios for various initial quaternion estimates. 50 MC runs. $\left(\sigma_{\epsilon },\sigma_{b}\right)=\left(0.1,0.1\right)$.

**Figure 3:**
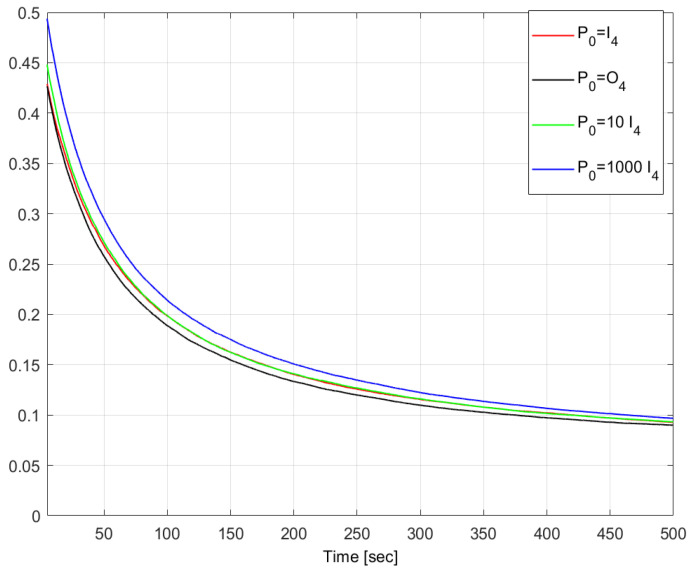
Time histories of the MC-mean of the Attenuation Ratios for various initial matrices $\tilde{P}\left(0\right)$. 50 MC runs. $\left(\sigma_{\epsilon },\sigma_{b}\right)=\left(0.1,0.1\right)$.

In a nutshell, the performance index AR(500) is not sensitive to variations in $\sigma_{\epsilon },\sigma_{b}$ below a threshold of $10^{-2}$, above which it decreases rapidly, thus, showing an improved performance in terms of disturbance attenuation. A similar lack of sensitivity is observed for the parameter $\gamma_{QHF}^{2}$ over the full ranges of $\sigma_{\epsilon }$ and $\sigma_{b}$, with a small but consistent improvement towards large values. Strikingly, the values of AR(500) are significantly lower than those of $\gamma_{QHF}^{2}$. In more detail, the gap is about six-fold lower in the case of vanishing variances, and about 30 times lower for very large variances, when the pair $\left(\sigma_{\epsilon },\sigma_{b}\right)$ is equal to (0.1,0.1). This is consistent with the $H_{\infty }$ filtering theory, i.e., vanishing disturbances are the worst case in terms of disturbance attenuation. The time variations of the MC averages of AR(t) and $\gamma_{QHF}^{2}\left(t\right)$ are depicted in Figure 1 for 0≤t≤2000 s, showing that the gap between them is already large from the start. The properties of the filter are further investigated in Figure 2 and Figure 3, which depict the time variations of AR for various initial estimates of the quaternion, $\hat{q}\left(0\right)$, and various initial values of the matrix $\tilde{P}\left(0\right)$, respectively. This is done for the case of $\left(\sigma_{\epsilon },\sigma_{b}\right)=\left(0.1,0.1\right)$, where the disturbance attenuation performance is best. It appears that the transient of AR is strongly shortened when $\hat{q}\left(0\right)$ is close to the true quaternion. On the other hand, the steady states are relatively close. Figure 3 further shows the lack of sensitivity of AR to $\tilde{P}\left(0\right)$. These properties stem from the independence of the estimator’s gain computations from the state and are analogous to the convergence properties of covariances in Kalman filters for linear systems.

**Figure 4:**
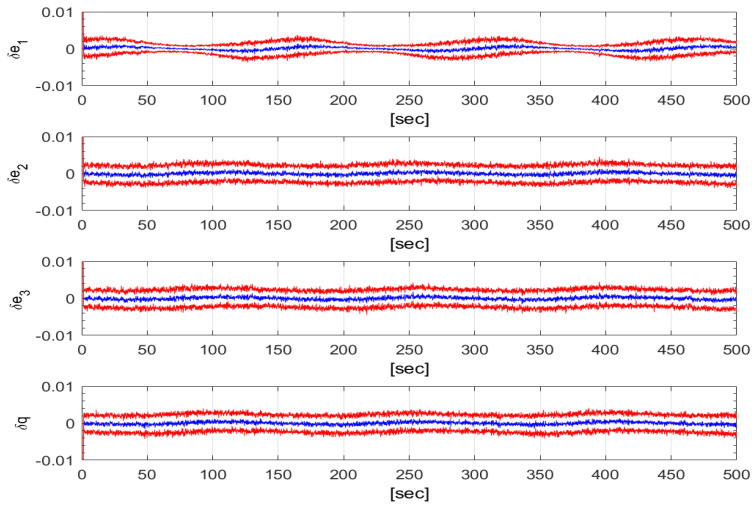
Time histories of the quaternion estimation error MC-means (blue) and MC-standard deviations (red). 50 MC runs. $\left(\sigma_{\epsilon },\sigma_{b}\right)=\left(0.001,0.1\right)$.

**Figure 5:**
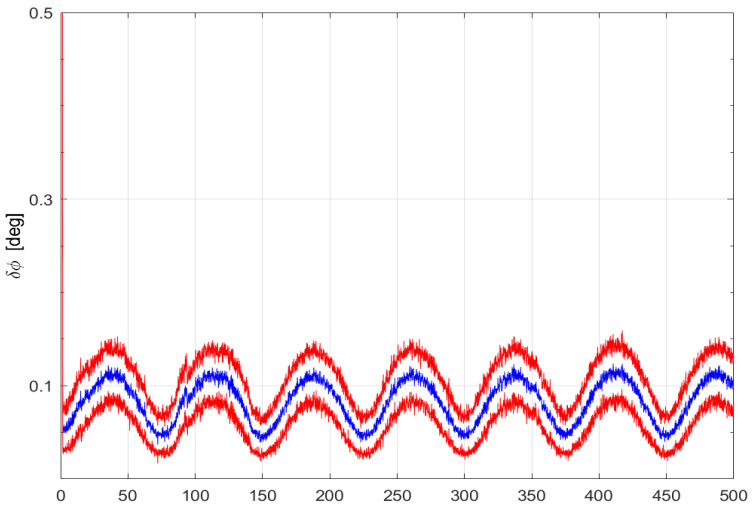
Time histories of the angular estimation error MC-mean (blue) and of the ± MC-σ envelope (red). 50 MC runs. $\left(\sigma_{\epsilon },\sigma_{b}\right)=\left(0.001,0.1\right)$.

Figure 4 depicts the MC-means and the MC-standard deviations of the four components of the quaternion estimation error for $\sigma_{\epsilon }=0.001\frac{\mathrm{rad}}{\sqrt{s}}$ and $\sigma_{b}=0.1$ rad. The means are close to zero and the standard deviations show satisfying estimation performances, around 3 mrad. Figure 5 presents the time histories of the MC-mean and MC-standard deviation envelop of the angular estimation error, δϕ. Albeit oscillating with an amplitude of 0.06[deg] around 0.08[deg], δϕ shows good performances given the measurement noise level $\sigma_{b}$ of about 5 degrees.

Extensive simulations were run to compare the performances of the QHF with those of a quaternion multiplicative EKF (MEKF) and an unscented quaternion filter (UQF) [5]. In the MEKF, the (quadratic in $q_{t}$) measurement equation model is linearized, and the filter statistics are matched to the true noise levels. In the UQF, we picked the design parameter λ=2 and the Generalized Rodriguez Parameters offset and scaling to be 0 and 1, respectively. Table 4 shows the Monte Carlo averages, computed on 50 runs of 2000 s, of the quaternion additive estimation error norm in the UQF (left), MEKF (center), and QHF (right). These values provide sensible measures for the estimation biases. In addition, the Monte Carlo standard deviations are provided for the three filters (in parenthesis). The QHF consistently provides smaller biases than the MEKF and the UQF. The latter usually exhibits smaller biases than the MEKF. This is explained by the linearization effects impairing the MEKF, whereas the QHF and the UQF avoid linearization. Yet, the UQF and the QHF implement different measurement equations. The UQF measurement is quadratic in $q_{t}$ while the QHF measurement model is linear in $q_{t}$. On the other hand, the MEKF provides smaller standard deviations than the QHF, as expected as the MEKF is a (approximate) minimum variance estimator. The UQF provides smaller variances than the MEKF, thanks to its unique methodology in calculating the optimal gain. Table 4 shows that, for a given value of $\sigma_{b}$, the gap between the filters decreases as $\sigma_{\epsilon }$ increases and becomes negligible for large $\sigma_{\epsilon }$. Table 4 further shows the low sensitivity of the QHF standard deviations as a function of the parameters $\sigma_{\epsilon }$ and $\sigma_{b}$. The $H_{\infty }$ approach partially explains this property as the gain computations are independent of $\sigma_{b}$ and $\sigma_{\epsilon }$ per se.

**Figure 6:**
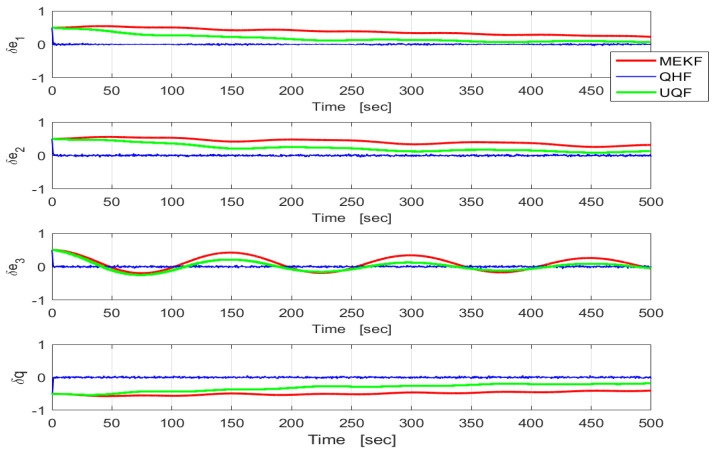
Time histories of the MC-means of the quaternion estimation errors in QHF (blue), MEKF (red), and UQF (green). Case A. 50 MC runs. $\left(\sigma_{\epsilon },\sigma_{b}\right)=\left(0.001,0.1\right)$.

**Figure 7:**
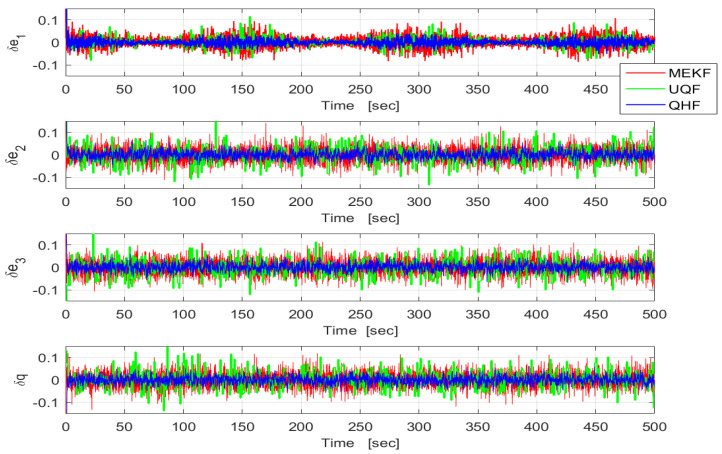
Time histories of the MC-means of the quaternion estimation errors in QHF (blue), MEKF (red), and UQF (green). Case B. 50 MC runs. $\left(\sigma_{\epsilon },\sigma_{b}\right)=\left(0.001,0.1\right)$.

The three filters were tested in cases where the true noise variances were unknown. This might occur as a result of undetected sensor failures or jamming. In Case A, $\sigma_{b}$ in UQF and MEKF was set to 10 times its true value. In Case B, it was lowered to one-tenth of the true value. The results are shown in Figure 6 and Figure 7, respectively. In case A, UQF and MEKF are very slow to converge while in case B they converge quicker but to noisier steady states. The QHF, on the other hand, provides essentially the same performances in both cases, with slight variations due to the data randomness. In both cases, the QHF outperforms the unmatched filters.

## 5. Conclusions

In this work, stochastic $H_{\infty }$ filters are developed to estimate the attitude quaternion from rate gyro and vector measurements. The cases of gyro white noise and gyro biases driven by white noise are considered. A key assumption is that the variances of all white noises are treated as disturbances. The estimators compute the quaternion while attenuating the transmission from the noise variances to the estimation error. The $H_{\infty }$ filters require solving a set of differential and algebraic linear matrix inequalities. A remarkable property of the resulting gains computations is that they are independent of the estimated quaternion in the case of null gyro drift. Extensive Monte-Carlo simulations show that the proposed filter performs well from the standpoint of attitude estimation per se in a wide range of gyro and vector observations variances. The guaranteed disturbance attenuation level appears slightly dependent on the variances as the gain is a function of the measurements. The actual disturbance attenuation level is better than the guaranteed one by up to one order of magnitude. It improves when the noise level increases and is the worst for (ideal) noise-free sensors. This agrees with the theory and illustrates the conservative nature of the $H_{\infty }$ approach. Extensive Monte-Carlo simulations show that QHF exhibits smaller biases in general compared to standard nonlinear optimal filters, like a multiplicative quaternion Kalman filter or an unscented quaternion filter. The matched unscented and Kalman filters exhibit lower standard deviations than the $H_{\infty }$ filter. But the higher the level of the noise, the lesser the advantage of the nonlinear optimal filters. Furthermore, the $H_{\infty }$ filter gain is less sensitive than the MEKF or UQF gain to perturbations like initial estimation errors. This is demonstrated by comparing the $H_{\infty }$ filter’s performances with those of unmatched MEKF and UQF. The unmatched MEKF and UQF were outperformed by the $H_{\infty }$ filter, which delivers identical performances within a wide range of noise variances.

## Figures and Tables

**Table 1:** QHF performance. Maxima of MC-means of $\delta_{q}$ and of $\frac{\delta q}{\sigma_{\epsilon }}$ for various $\sigma_{\epsilon }$. 500 s, 500 runs.

$\sigma_{\epsilon }\;\;\;\left[\frac{\mathrm{rad}}{\sqrt{s}}\right]$	$10^{-4}$	$10^{-3}$	$10^{-2}$	$10^{-1}$	$10^{0}$
$\delta_{q}$	$2.5\times 10^{-5}$	$2.2\times 10^{-4}$	$1.7\times 10^{-3}$	$1.2\times 10^{-2}$	$6.2\times 10^{-2}$
$\frac{\delta q}{\sigma_{\epsilon }\sqrt{\Delta t}}$	0.79	0.69	0.53	0.37	0.18

**Table 2:** Ratios of the δϕ MC-means of the QHF over the QKF for various $\sigma_{\epsilon }$ and $\sigma_{b}$. (Time-average of the δϕ MC-mean in the QHF in degree). 500 runs, 6000 s.

$\frac{\sigma_{\epsilon }\left[\frac{\mathrm{rad}}{\sqrt{s}}\right]}{\sigma_{b}\left[\mathrm{rad}\right]}$	$10^{-4}$	$10^{-3}$	$10^{-2}$	$10^{-1}$	$10^{0}$
$10^{-5}$	$\overset{\left(1.4\;\times \;10^{-3}\right)}{0.51}$	$\overset{\left(1.8\;\times \;10^{-2}\right)}{0.15}$	$\overset{\left(6.6\;\times \;10^{-2}\right)}{0.08}$	$\overset{\left(7.2\;\times \;10^{-1}\right)}{0.02}$	$\overset{\left(3.4\;\times \;10^{0}\right)}{0.01}$
$10^{-4}$	$\overset{\left(6.4\;\times \;10^{-3}\right)}{0.65}$	$\overset{\left(5.2\;\times \;10^{-2}\right)}{0.52}$	$\overset{\left(2.7\;\times \;10^{-1}\right)}{0.18}$	$\overset{\left(9.5\;\times \;10^{-1}\right)}{0.07}$	$\overset{\left(6.1\;\times \;10^{0}\right)}{0.02}$
$10^{-3}$	$\overset{\left(1.9\;\times \;10^{-2}\right)}{1.18}$	$\overset{\left(8.6\;\times \;10^{-2}\right)}{0.63}$	$\overset{\left(5.1\;\times \;10^{-1}\right)}{0.49}$	$\overset{\left(1.6\;\times \;10^{0}\right)}{0.17}$	$\overset{\left(6.4\;\times \;10^{0}\right)}{0.09}$

**Table 3:** Attenuation Ratios AR(500) (500 MC runs) as defined in Equation (93) for various values of the parameters $\left\{\sigma_{\epsilon },\sigma_{b}\right\}$. (Value of the steady-state MC-mean of $\gamma_{QHF}^{2}$, as computed in the filter).

$\frac{\sigma_{\epsilon }\left[\frac{\mathrm{rad}}{\sqrt{s}}\right]}{\sigma_{b}\left[\mathrm{rad}\right]}$	0	$10^{-3}$	$10^{-2}$	$2\;\times \;10^{-2}$	$5\;\times \;10^{-2}$	$10^{-1}$
**0**	$\overset{\left(2.89\right)}{0.45}$	$\overset{\left(2.89\right)}{0.45}$	$\overset{\left(2.79\right)}{0.44}$	$\overset{\left(2.56\right)}{0.21}$	$\overset{\left(2.43\right)}{0.13}$	$\overset{\left(2.32\right)}{0.10}$
$10^{-3}$	$\overset{\left(2.89\right)}{0.45}$	$\overset{\left(2.65\right)}{0.45}$	$\overset{\left(2.60\right)}{0.44}$	$\overset{\left(2.53\right)}{0.21}$	$\overset{\left(2.40\right)}{0.11}$	$\overset{\left(2.32\right)}{0.09}$
$10^{-2}$	$\overset{\left(2.78\right)}{0.44}$	$\overset{\left(2.53\right)}{0.43}$	$\overset{\left(2.46\right)}{0.41}$	$\overset{\left(2.42\right)}{0.18}$	$\overset{\left(2.36\right)}{0.10}$	$\overset{\left(2.31\right)}{0.09}$
$5\;\times \;10^{-2}$	$\overset{\left(2.55\right)}{0.31}$	$\overset{\left(2.44\right)}{0.30}$	$\overset{\left(2.40\right)}{0.28}$	$\overset{\left(2.39\right)}{0.16}$	$\overset{\left(2.33\right)}{0.10}$	$\overset{\left(2.31\right)}{0.09}$
$10^{-1}$	$\overset{\left(2.41\right)}{0.16}$	$\overset{\left(2.40\right)}{0.15}$	$\overset{\left(2.39\right)}{0.15}$	$\overset{\left(2.35\right)}{0.10}$	$\overset{\left(2.28\right)}{0.09}$	$\overset{\left(2.25\right)}{0.06}$

**Table 4:** Monte Carlo averages (and Monte Carlo standard deviations) of the quaternion estimation errors in the UQF, MEKF, and QHF for various values of the parameters $\left\{\sigma_{\epsilon },\sigma_{b}\right\}$. 50 runs. Time span of 2000 s.

$\sigma_{b}\left[\mathrm{rad}\right]$	$10^{-3}$	$10^{-2}$	$10^{-1}$
$\sigma_{\epsilon }\left[\frac{\mathrm{rad}}{\sqrt{s}}\right]$	UQF | MEKF | QHF	UQF | MEKF | QHF	UQF | MEKF | QHF
$10^{-7}$	$\overset{\left(1.0\times 10^{-5}\left|1.2\times 10^{-5}\right|1.4\times 10^{-3}\right)}{3\times 10^{-5}\left|3\times 10^{-5}\right|2\times 10^{-5}}$	$\overset{\left(9.0\times 10^{-5}\left|1.0\times 10^{-4}\right|1.4\times 10^{-3}\right)}{0.0003|0.0003|0.0003}$	$\overset{\left(0.013|0.015|0.080\right)}{0.017|0.020|0.014}$
$10^{-6}$	$\overset{\left(1.0\times 10^{-4}\left|1.2\times 10^{-4}\right|1.4\times 10^{-3}\right)}{4\times 10^{-5}\left|4\times 10^{-5}\right|2\times 10^{-5}}$	$\overset{\left(5.0\times 10^{-5}\left|8.0\times 10^{-5}\right|1.3\times 10^{-3}\right)}{0.0004|0.0005|0.0003}$	$\overset{\left(0.013|0.015|0.080\right)}{0.017|0.020|0.014}$
$10^{-5}$	$\overset{\left(1.5\times 10^{-4}\left|2.1\times 10^{-4}\right|1.4\times 10^{-3}\right)}{3\times 10^{-5}\left|5.1\times 10^{-5}\right|2.3\times 10^{-5}}$	$\overset{\left(7.0\times 10^{-4}\left|9.0\times 10^{-4}\right|1.4\times 10^{-3}\right)}{0.0008|0.0008|0.0007}$	$\overset{\left(0.012|0.015|0.080\right)}{0.019|0.020|0.016}$
$10^{-4}$	$\overset{\left(4.2\times 10^{-4}\left|6.2\times 10^{-4}\right|1.4\times 10^{-3}\right)}{5\times 10^{-5}\left|5.5\times 10^{-5}\right|2.5\times 10^{-5}}$	$\overset{\left(1.5\times 10^{-3}\left|2.0\times 10^{-3}\right|2.1\times 10^{-3}\right)}{0.0009|0.0011|0.0007}$	$\overset{\left(0.010|0.015|0.082\right)}{0.019|0.020|0.015}$
$10^{-3}$	$\overset{\left(1.0\times 10^{-3}\left|1.2\times 10^{-3}\right|1.4\times 10^{-3}\right)}{5.3\times 10^{-5}\left|7.9\times 10^{-5}\right|2.6\times 10^{-5}}$	$\overset{\left(2.8\times 10^{-3}\left|3.7\times 10^{-3}\right|1.2\times 10^{-2}\right)}{0.0015|0.0034|0.0008}$	$\overset{\left(0.013|0.015|0.081\right)}{0.019|0.020|0.016}$
$10^{-2}$	$\overset{\left(1.0\times 10^{-3}\left|1.6\times 10^{-3}\right|2.0\times 10^{-3}\right)}{7.3\times 10^{-5}\left|9.3\times 10^{-5}\right|2.8\times 10^{-5}}$	$\overset{\left(0.009|0.0120|0.0124\right)}{0.0022|0.0040|0.0014}$	$\overset{\left(0.016|0.019|0.080\right)}{0.020|0.020|0.016}$
$10^{-1}$	$\overset{\left(1.0\times 10^{-2}\left|1.6\times 10^{-2}\right|1.6\times 10^{-2}\right)}{8.1\times 10^{-5}\left|1.1\times 10^{-4}\right|3.3\times 10^{-5}}$	$\overset{\left(0.014|0.017|0.019\right)}{0.0025|0.0054|0.0017}$	$\overset{\left(0.028|0.035|0.084\right)}{0.040|0.040|0.017}$

## Data Availability

The original contributions presented in the study are included in the article, further inquiries can be directed to the corresponding author.
